# 

**Published:** 1924-01

**Authors:** 


### THE BRISTOL
### Medico-Chirurgical Journal.

A JOURNAL OF THE MEDICAL SCIENCES FOR THE WEST OF ENGLAND AND SOUTH WALES.

*PUBLISHED UNDER THE AUSPICES OF*

*THE BRISTOL MEDICO-CHIRURGICAL SOCIETY.*

EDITOR:
P. WATSON-WILLIAMS, M.D.

WITH WHOM ARE ASSOCIATED

JAMES SWAIN, C.B., C.B.E., M.S., M.D., J. LACY FIRTH, M.S., M.D.,
CAREY COOMBS, M.D.

J. A. NIXON, C.M.G., M.D., ASSISTANT EDITOR.

EDITORIAL SECRETARY:
A. L. FLEMMING, M.B., Ch.B.

"Scire est nescire, nisi id me

Scire alius scierit."

*VOL. XLI.*

Library of Congress

FEB 2 0 1925

545 880

Smithsonian Deposit

BRISTOL: J. W. ARROWSMITH LTD.
LONDON: J. W. ARROWSMITH (LONDON) LTD.
1924.

R31
.B8

### CONTENTS OF VOL. XLI.

*Page*

The Evolutionary History of Renal Surgery and of Temporal Bone Surgery. By J. Lacy Firth, M.D., M.S. Lond., F.R.C.S. .. 1

Duodenal Ulcers: their Detection by Photography. By T. Carwardine, M.S. .. .. .. .. .. .. .. 16

The Leprosy Problem and its Fearing on Tuberculosis. By Sir Leonard Rogers, C.I.E., M.D., F.R.C.P., F.R.C.S., F.R.S., I.M.S. Ret. .. .. .. .. .. .. .. .. 19

The Parkinsonian Syndrome; some points in Diagnosis and Treatment. By Macdonald Critchley, M.B., Ch.B. .. .. .. .. 25

The Evolutionary History of Renal Surgery, etc. Part II. .. .. 49

Cancer of the Breast. By A. Rendle Short, M.D., B.S., B.Sc., F.R.C.S. .. .. .. .. .. .. .. .. 64

Grave Cardiac Symptoms due to Multiple Dental Abscess. By W. R. Ackland, M.D.S. .. .. .. .. .. .. 79

The Therapeutic Uses of Radium. By A. E. Hayward Pinch, F.R.C.S. .. .. .. .. .. .. .. .. 97

Diverticulitis of the Pelvic Colon. By Sir C. Gordon-Watson, K.B.E., C.M.G., F.R.C.S. .. .. .. .. .. .. 112

Recovery after Mechanical Obstruction due to the Passage of a Gallstone through the Intestine, with Septic Peritonitis. By C. A. Morton, F.R.C.S. .. .. .. .. .. 126

Labyrinthitis. By E. Watson-Williams, M.C., Ch.M. .. .. 135

The Long Fox Lecture: The Conditioned Response. By C. Lloyd Morgan, LL.D., D.Sc., F.R.S. .. .. .. .. .. 165

Auricular Failure. By Carey F. Coombs, M.D., F.R.C.P. Lond., and C. E. K. Herapath, M.C., M.D. Lond. .. .. .. 180

A Few Suggestions in General Surgery. By C. F. Walters, F.R.C.S. 202

Reviews of Books .. .. .. .. .. 34, 82, 141, 210

Editorial Notes .. .. .. .. .. .. 41, 87, 148, 214

Meetings of Societies .. .. .. .. .. .. 42, 88, 156

The Library of the Bristol Medico-Chirurgical Society 45, 162, 215

Obituary .. .. .. .. .. .. .. .. 47, 89, 157, 213

Local Medical Notes .. .. .. .. .. 48, 95, 163, 216

WAC 10/28/42

### Bristol
### Medico-Chirurgical Journal.

Published Quarterly (demy 8vo)
under the auspices of the Bristol
Medico-Chirurgical Society.

*An excellent means of advertising*

The only Medical Journal published in the West of England, and has an extensive circulation not only amongst the Medical men of Bristol, but also amongst those of the adjoining Counties and South Wales, and in exchange goes to all parts of the world.

Terms for Advertisements:
Whole Page, £2 2s.; Half Page, £1 5s.; Quarter Page, 15s.
per issue.

PRICES FOR SPECIAL POSITIONS ON APPLICATION.

J. W. ARROWSMITH LTD.,

Advertising Agents,

11 Quay Street,

BRISTOL.

SPECIMEN

JOURNAL

SENT ON

REQUEST.

### Bristol Medico-Chirurgical Society.

### President.

J. LACY FIRTH, M.D., M.S. Lond.

### President-Elect.

J. ODERY SYMES, M.D. Lond.

### Committee.

*Ex-Officio*:
J. A. NIXON, *C.M.G.*, B.A., M.D.Cantab. (Retiring President).
P. WATSON-WILLIAMS, M.D. Lond. (Editor of *Journal*).
C. K. RUDGE (Hon. Librarian).
A. L. FLEMMING, M.B., Ch.B. Brist. (Editorial Secretary).

*Elected.*

F. LACE } Ex-Presidents.

R. G. P. LANSDOWN, M.D., B.S. Durh. } Ex-Presidents.

T. CARWARDINE, M.S. Lond.

D. C. RAYNER.

FORBES FRASER.

C. FERRIER WALTERS.

E. W. HEY GROVES, M.D., M.S. Lond.

A. J. WRIGHT, M.B., B.S. Lond.

### Honorary Secretary.

S. V. STOCK, M.B., B.S. Lond.

Medical Practitioners wishing to join the Society are requested to communicate with the Hon. Secretary, Mr. S. V. STOCK, 1 Mortimer Road, Clifton. The Annual Subscription is £1 11s. 6d.

---

*Extracts from Journal By-laws.*

4.—The *Journal* shall be delivered free to Subscribers and also to Members of the Society whose subscriptions are not in arrear.

6.—The Annual Subscription to the *Journal* is 10/6.

---

Communications intended for insertion in the *Journal* should be sent to the Editor, Dr. P. WATSON-WILLIAMS, 2 Rodney Place, Clifton, Bristol.

Books for Review and Exchange Journals should be sent to the Assistant-Editor, *Bristol Medico-Chirurgical Journal*, Medical Library, The University, Bristol.

Communications referring to the delivery of the *Journal* should be sent to the Editorial Secretary, A. L. Flemming, 48 Pembroke Road, Clifton, Bristol, to whom Subscriptions are to be paid *in advance*.

Cases for Binding Vols. I—XL are now ready, and may be obtained, price 1/9 each (postage extra), upon application to J. W. ARROWSMITH LTD., Quay Street, Bristol; or the *Journal* will be bound, including Case, for 7/- per volume.

### Bristol Medico-Chirurgical Society.

### PAST PRESIDENTS.

1874—75. FREDERICK BRITTAN, M.D., B.A. Dub.
1875—76. ROBERT WILLIAM COE.
1876—77. SAMUEL MARTYN, M.D. Ed.
1877—78. AUGUSTIN PRICHARD, M.D. Berlin.
1878—79. HENRY EDWARD FRIPP, M.D. Würzburg.
1879—80. WILLIAM MICHELL CLARKE.
1880—81. JOSEPH GRIFFITHS SWAYNE, M.D. Lond.
1881—82. EDWARD LONG FOX, M.D. Oxon.
1882—83. JAMES GEORGE DAVEY, M.D. St. And.
1883—84. WILLIAM JOHNSTONE FYFFE, M.D., B.A. Dub.
1884—85. GEORGE FORSTER BURDER, M.D. Aberd.
1885—86. WILLIAM HENRY SPENCER, M.D., M.A. Cantab.
1886—87. FRANCIS POOLE LANSDOWN.
1887—88. LEMUEL MATTHEWS GRIFFITHS.
1888—89. ROBERT SHINGLETON SMITH, M.D., B.Sc. Lond.
1889—90. NELSON CONGREVE DOBSON, Ch.M. Bristol.
1890—91. SAMUEL HENRY SWAYNE.
1891—92. FRANCIS RICHARDSON CROSS, M.B.Lond., LL.D.Bristol.
1892—93. EDWARD MARKHAM SKERRITT, M.D., B.S., B.A. Lond.
1893—94. JAMES GREIG SMITH, M.B., C.M., M.A. Aberd., F.R.S.E.
1894—95. ALFRED JAMES HARRISON, M.B. Lond.
1895—96. ARTHUR WILLIAM PRICHARD.
1896—97. ALFRED EDWARD AUST LAWRENCE, M.D., C.M. Aberd.
1897—98. JOHN EDWARD SHAW, M.B., C.M. Ed.
1898—99. ROBERT ROXBURGH, M.B. Ed.
1899—00. WILLIAM HENRY HARSANT.
1900—01. DAVID SAMUEL DAVIES, M.D., Lond.
1901—02. BARCLAY JOSIAH BARON, M.B., C.M. Ed.
1902—03. GEORGE MUNRO SMITH, M.D. Bristol.
1903—04. JAMES PAUL BUSH, C.M.G., C.B.E., Ch.M. Bristol.
1904—05. REGINALD EAGER, M.D. Lond.
1905—06. JOHN DACRE.
1906—07. JAMES TAYLOR.
1907—08. HENRY WALDO, M.D., C.M. Aberd.
1908—09. JOHN MICHELL CLARKE, M.D., M.A. Cantab.
1909—10. JAMES SWAIN, C.B., C.B.E., M.D., M.S. Lond.
1910—11. BERTRAM MITFORD HERON ROGERS, M.D., B.Ch., B.A. Oxon.
1911—12. CHARLES ALEXANDER MORTON.
1912—13. WALTER CARLESS SWAYNE, M.D., B.S. Lond. and Bristol.
1913—14. PATRICK WATSON-WILLIAMS, M.D. Lond.
1914—15. WILLIAM HARRY CHRISTOPHER NEWNHAM, M.A., M.B. Cantab.
1915—16 GEORGE PARKER, M.A., M.D. Cantab.
1916—17 FRANCIS HENRY EDGEWORTH, M.A., M.D. Cantab., D.Sc. Lond.
1917—18 FREDERICK LACE.
1918—19 ROBERT GUTHRIE POOLE LANSDOWN, M.D., B.S. Durh.
1919—20 I. WALKER HALL, M.D., Ch.B. Vict.
1920—21 LEOPOLD ERNEST VALENTINE EVERY-CLAYTON, M.D. Lond.
1921—22 CYRIL HUTCHINSON WALKER, M.B., B.A. Cantab.
1922—23 JOHN ALEXANDER NIXON, C.M.G., B.A., M.D. Cantab.

1924.

### LIST OF SUBSCRIBERS

TO THE

### Bristol Medico-Chirurgical Journal.

### HONORARY MEMBERS OF THE SOCIETY.

| | |
|---|---|
| Griffiths, L. M. ... ... ... ... ... | 11 Pembroke Road, Clifton. |
| Owen, Sir Isambard, D.C.L., M.D. | Abbey Gate, Beach Road, Weston-super-Mare. |
| Rudge, C. King ... ... ... ... ... ... | 145 Whiteladies Rd., Clifton. |

### SUBSCRIBERS.

*Those marked * are Members of the Society.*

| | |
|---|---|
| *Adams, A. W., M.B., B.Sc. Lond. ... ... ... | 9 Mortimer Road, Clifton. |
| Adye, W. J. A. ... ... ... .. ... ... ... | St. Margarets, Bradford-on Avon, Wilts. |
| *Alexander, A., M.A. ... ... ... ... ... .. | 23 Manilla Road, Clifton. |
| *Alexander, D. A., M.B., Ch.B. Vict. ... ... | 30 Berkeley Square, Clifton. |
| *Askins, R. A., M.D., B.Ch., B.A.O. Dubl.... | Guildhall, Bristol. |
| *Aubrey, T., M.B. Lond. ... ... ... ... ... | Bitton, near Bristol. |
| *Baker, Lily A., M.D., B.Ch., B.A.O., R.U.I.... | 21 Victoria Square, Clifton. |
| *Barber, M. C., M.B., Ch.B. Brist. ... ... ... | Ingledene, High Street, Staple Hill, Bristol. |
| *Barker, G. H., M.D., B.Sc. Lond. ... ... ... | 124 Redland Road, Bristol. |
| *Bartholomew, E. U. ... ... ... ... ... ... | 12 Lansdown Place, Clifton. |

### LIST OF SUBSCRIBERS.

| | |
|---|---|
| *BERGIN, F. G. ... ... ... ... ... ... ... ... | 31 Oakfield Road, Clifton. |
| BERNARD, C. ... ... ... ... ... ... ... ... ... | 704 Fishponds Road, Fishponds, Bristol |
| BERRY, F. C., M.D., B.Ch., B.A. Dub. ... ... | Locarno, Burnham, Somerset. |
| *BIRRELL, J. A., M.B., B.S. Lond. ... ... | 28 Berkeley Square, Clifton. |
| *BLACKER, A. E.... ... ... ... ... ... ... ... | 20 Victoria Square, Clifton. |
| BLACKETT, J. F., M.D., B.S. Lond. ... ... ... | Hamsterley, Bloomfield Park, Bath. |
| *BLAGG, ARTHUR F., M.D. Durh.... ... ... ... | 28 Caledonia Place, Clifton. |
| *BODMAN, C. O., M.D. Durh. ... ... ... ... | 17 Upper Belgrave Road, Clifton. |
| *BODMAN, J. HERVEY, M.D. Lond. ... ... ... | 9 Whiteladies Road, Clifton. |
| *BOWKER, G. E., M.D. Edin.... ... ... ... ... | 11 North Parade, Bath. |
| *BRAY, G., Lt.-Col., *D.S.O.* ... ... ... | 10 Miles Road, Clifton. |
| *BRISTOWE, H. C., M.D. Lond. ... ... ... ... | Wrington, Somerset. |
| BROWN, WILLIAM, M.D., C.M. Glasg. ... ... | Park View, Fishponds, Bristol. |
| *BUCKMASTER, G. A., M.A., M.D. Oxon. ... ... | University of Bristol. |
| *BURDON-COOPER, J., M.D., B.Sc. Durh. ... ... | 12 The Circus, Bath. |
| *BUSH, J. PAUL, *C.M.G.*, *C.B.E.*, Ch.M. Brist. | Vyvyan House, Clifton. |

| | |
|---|---|
| *CAIRNS, F. J. ... ... ... ... ... ... ... ... | Devon House, Whitehall Road, Bristol. |
| *CAREY, R. S., *O.B.E.*, B.A. Cantab. ... ... | 502 Bath Road, Brislington, Bristol. |
| *CARLETON, H. H., M.A., M.D. B.Ch. Oxon. | 72 Pembroke Road, Clifton. |
| *CARLING, A., M.A., M.B., B.C. Cantab. ... ... | 12 Great George Street, Bristol. |
| *CARWARDINE, THOMAS, M.B., M.S. Lond. ... | 16 Victoria Square, Clifton. |
| *CAVE, EDWARD J., M.D. Lond. ... ... ... ... | 20 The Circus, Bath. |
| *CHAMBERS, E. R. ... ... ... ... ... ... | 9 Clifton Park, Clifton. |
| *CHARLES, J. R., M.A., M.D., B.C. Cantab. ... | Deepholm, Clifton. |
| *CHITTY, H., M.S. Lond. ... ... ... ... ... | 46 Pembroke Road, Clifton. |
| *CHRISTOFFERSON, P. E., M.B., Ch.B. Brist. ... | 8 Lansdown Place, Clifton. |
| *CLAREMONT, L. E. ... ... ... ... ... ... | 5 Rodney Place, Clifton. |
| *CLARKE, CECIL, M.B., B.S. Lond. ... ... ... | Brook House, Axmouth, Devon. |
| *CLARKE, R. C., *O.B.E.*, M.B., Ch.B. Brist. ... | 29 Victoria Square, Clifton. |
| *COATES, VINCENT M., *M.C.*, M.A., M.D B.C. Cantab. .. ... ... ... ... ... ... | 10 The Circus, Bath. |
| CONNELLAN, P. S. ... ... ... ... ... ... ... | 12 Bloomsbury Street, London, W.C.1. |
| *COOMBS, CAREY, M.D. Lond. ... ... ... ... | 3 Pembroke Road, Clifton. |
| *COOPER, WILLIAM RUSSELL ... ... ... ... | 13 Westfield Park, Redland, Bristol. |
| *CORFIELD, C., M.D. Durh. ... ... ... ... | 5 Kensington Place, Clifton. |
| COSSHAM, W. R., M.D., C.M. Aberd.... ... ... | Wellesley House, Cirencester. |
| CRIDLAND, A. B. ... ... ... ... ... ... ... | Salisbury House, Chapel Ash, Wolverhampton. |
| *CROSS, F. R., M.B. Lond., LL.D. Brist. ... | Worcester House, Clifton. |
| *CROSSMAN, F. W., M.B. Durh. ... ... ... ... | White's Hill, Hambrook near Bristol. |

| | |
|---|---|
| *DACRE, JOHN ... ... ... ... ... ... ... ... | 14 Eaton Crescent, Clifton. |
| *DAVIES, E. D. D. ... ... ... ... ... ... ... | 6 Lansdown Place, Clifton. |
| *DAVIES, D. S., M.D. Lond., D.P.H. Cantab. ... | 6 Lansdown Place, Clifton. |
| *DAWE, J. H., M.B. Edin. ... ... ... ... ... | North Parade House, Bath. |

### LIST OF SUBSCRIBERS.

| | |
|---|---|
| DENING, EDWIN ... ... ... ... ... ... ... | Manor House, Stow-on-the-Wold, Glos. |
| DEVINE, H., M.D Lond. ... ... ... ... ... | Corporation Mental Hospital, Portsmouth. |
| *DEVIS, H. F. ... ... ... ... ... ... ... ... ... | 160 Wells Road, Bristol. |
| *DIX, C.... ... ... ... ... ... ... ... ... ... ... | 11 Victoria Square, Clifton. |
| *DIXON, G. C., B.A., M.B. ... ... ... ... ... | Bristol Eye Hospital. |
| *DIXON, T. B. ... ... ... ... ... ... ... ... | 134 Coronation Road, Bristol. |
| *DRAPES, T. L., M.B., B.C., B.A. Cantab. ... | St. Maur, Beaufort Square, Chepstow. |
| DYMOKE, F. ... ... ... ... ... ... ... ... | 21 Cotham Road, Bristol. |

| | |
|---|---|
| *EDGEWORTH, F. H., M.D., B.C., M.A. Cantab., D.Sc. Lond. ... ... ... ... ... ... ... | 13 Richmond Hill, Clifton. |
| EDWARDS, C. D., B.A., M.D. Cantab. ... ... | The Corderies, Chalford, Glos. |
| *EGLINGTON, J. H. C. ... ... ... ... ... ... | Street, Somerset. |
| *ELKINGTON, G. W. ... ... ... ... .. ... | 29 Victoria Square, Clifton. |
| ELLIOT BROS. LTD. ... ... ... ... ... ... | *Australian Pharmaceutical Notes & News*, O'Connell Street, Sydney, N.S.W. |
| ELLIOTT, F. PERCY, M.B., C.M. Aberd. ... ... | 113 Grove Rd., Walthamstow, London, E.17. |
| *ELLIOTT, W. H. A., M.B., B.S. Lond. ... ... | 2 Oakfield Road, Clifton. |
| *EVERY-CLAYTON, L. E. V., M.D. Lond ... ... | The Old School House, Tetbury. |

| | |
|---|---|
| *FAILL, C. J. C. ... ... ... ... ... ... ... ... | 19 Portland Square, Bristol. |
| FARNFIELD, W. W.... ... ... .. .. ... ... | Clive Vale, Gillingham, Dorset. |
| *FALLON, Col. J. ... ... ... ... ... ... ... | 35 Cavendish Road, Henleaze, Bristol. |
| *FAWN, G. F. ... ... ... ... ... ... ... ... | 6 Oakfield Road, Clifton. |
| *FELLS, A., M.B., C.M. Edin. ... ... ... ... | 6 Elton Road, Clifton. |
| *FIRTH, J. L., M.D., M.S. Lond. ... ... ... ... | 8 Victoria Square, Clifton. |
| *FLEMMING, A. L., M.B., Ch.B. Brist. ... ... | 48 Pembroke Road, Clifton. |
| *FLEMMING C. E. S. ... ... ... ... ... ... | Manvers House, Bradford-on-Avon. |
| *FOSS, E. V., M.D., Ch.B. Brist. ... ... ... | Clouds Hill House, St. George, Bristol. |
| *FRASER, A. D., M.A., M.B., Ch.B. ... ... ... | 4 Alexandra Road, Clifton. |
| *FRASER, FORBES, *C.B.E.* ... ... ... ... ... | 5 The Circus, Bath. |

| | |
|---|---|
| *GARDEN, R. R., M.A., M.B., B.Ch. ... ... ... | Guildhall, Bristol. |
| GARLAND, E. B., M.B., C.M. Edin. ... ... ... | 114*a* Hampton Road, Redland Bristol |
| GEE, C. A. H., M.B., Ch.B. Brist. ... ... ... | Evercreech, Somerset. |
| *GERRISH, D. S. ... ... ... ... ... ... ... | 328 & 330 Church Road, St. George Bristol. |
| *GIBBS, A. N. GODBY ... ... ... ... ... ... | 52 Whiteladies Road, Clifton. |
| *GLENNY, E. T., M.B., B.S. Lond. ... ... ... | 102 Pembroke Road, Clifton |
| *GOLDING, MADGE E., M.B., Ch.B. ... ... ... | Bristol Royal Infirmary. |
| *GORDON, R. G. M.D., B.Sc. Edin. ... ... ... | 9 Circus, Bath. |
| *GREEN, T. A., *D.S.O.*, M.D., C.M. Edin. ... | 33*a* Whiteladies Road, Clifton |
| GRIFFITHS, CORNELIUS A. ... ... ... ... ... | 35 Newport Road, Cardiff. |
| *GRIFFITHS, J. S. ... ... .. ... ... ... ... | 20 Redland Park, Bristol. |
| *GROVES, E. W. HEY., M.D., M.S. Lond.... ... | 25 Victoria Square, Clifton. |

### LIST OF SUBSCRIBERS.

| | |
|---|---|
| *HADFIELD, G., M.D. Lond. ... ... ... ... | General Hospital, Bristol. |
| *HAILES CLEMENTS D. G., M.D., C.M. Ed. ... | 2 Richmond Park Road, Clifton. |
| *HALL, E. GEORGE, M.B. Lond. ... ... ... ... | 42 Apsley Road, Clifton, Bristol. |
| *HALL, I. WALKER, M.D., Ch.B.Vict. ... ... | Manilla Lodge, Clifton. |
| *HARRIS, H. ELWIN, B.A., M.B. Cantab. ... ... | 13 Lansdown Place, Clifton. |
| *HARSANT, W. H. ... ... ... ... ... ... ... | Tower House, Clifton Down Road, Clifton. |
| *HARTY, J. P. I., M.B., B.Ch., B.A.O., R.U.I. | Deepholm, Clifton. |
| *HAWKINS, E. J. ... ... ... ... ... ... ... | Constantine House, Avonmouth. |
| *HERAPATH, C. E. K., *M.C.*, M.D. Lond. ... ... | Ormlie, Clifton. |
| *HILL, HEDLEY, M.D. Durh. ... ... ... ... | 101 Pembroke Road, Clifton. |
| *HOLLAND, W. A. L. ... ... ... ... ... ... | Pensions Hospital, Bath. |
| HOSPITAL, BRISTOL GENERAL (*Secretary*, T. W. GREGG). | |
| *HUGHES, D. ... ... ... ... ... ... ... ... | 12 Cotham Road, Bristol. |

| | |
|---|---|
| *ILES, A. E., *O.B.E.*, M.B., B.S. Lond. ... ... | 17 Victoria Square, Clifton. |
| INFIRMARY, BRISTOL ROYAL (*Secretary*, E. C. SMITH). | |
| INGLE, C. DURBAN ... ... ... ... ... ... ... | Somerton, Somerset. |

| | |
|---|---|
| *JACKMAN, W. A. ... ... ... ... ... ... ... | 24 College Road, Clifton. |
| *JOHNSON, R. G., M.B., D.P.H. ... ... ... ... | 119 Redland Road, Bristol. |
| *JOLL, C. A., M.B., M.S. Lond. ... ... ... ... | 23 New Cavendish Street, W.1. |
| *JONES, J. ELLINGTON ... ... ... ... .. ... | 49 Fernbank Road, Redland. |

| | |
|---|---|
| *KENNEDY, W. P. M.D. Dubl. ... ... ... ... | 3 Gay Street, Bath. |
| *KERFOOT, STANLEY J. ... ... ... ... ... ... | 109 East Street, Bedminster, Bristol. |
| *KINGSTON, S. H., M.B., Ch.B. Brist. .. ... | 3 Whiteladies Road, Clifton. |
| *KYLE, H. G., M.A., M.D. B.Ch. Oxon. ... ... | 31 Westbury Road, Bristol. |

| | |
|---|---|
| *LACE, F. ... ... ... ... ... ... ... ... ... | 5 Gay Street, Bath. |
| *LANSDOWN, R. G. P., M.D., B.S. Durh. ... ... | 39 Oakfield Road, Clifton. |
| *LAVINGTON, C C., M.B., B.S. Durh. ... ... | 1 Burlington Road, Redland, Bristol. |
| *LEES, E. LEONARD, M.D., C.M. Ed. ... ... ... | 22 Richmond Hill, Clifton. |
| *LEGAT, R. EDDOWES M.B., C.M. Edin.... ... | Murray House Staple Hill, Bristol. |

### LIST OF SUBSCRIBERS.

| | |
|---|---|
| Leigh, W. W. ... ... ... ... ... ... ... ... | Treharris, Glamorganshire. |
| *Leonard, R. C., M.D. Durham ... ... ... ... | Bower Ashton, Bristol. |
| *Levis, J. S., *M.C.*, M.B., B.Ch., N.U.I. ... | 20 Gay Street, Bath. |
| *Lewis, D. J. ... ... ... ... ... ... ... ... | 11 Gt. Bedford Street, Bath. |
| *Linton, Marion S., M.B. Lond. ... ... ... | 21 Oakfield Road, Clifton. |
| *Lister, L. Margaret ... ... ... ... ... | 12 All Saints Road, Clifton. |
| *Logan, H. B. ... ... ... ... ... ... ... ... | Eastfield, Southville, Bristol. |
| *Lucas, J. J. S., M.D. Lond. ... ... ... ... ... | 186 Wells Road, Totterdown, Bristol. |

| | |
|---|---|
| Mackinnon, Charles, M.B., C.M., M.A. Glasg. | Davaar, Cirencester. |
| Maclean, Ewen J., M.D., C.M. Ed.... ... ... | 12 Park Place, Cardiff. |
| Maclean, J. Campbell, M.B., C.M. Ed.... ... | Swindon House, Swindon. |
| *MacLeod, Donald, M.B., C.M. Ed. ... ... | 84 Redland Road, Redland, Bristol. |
| *MacLeod, G., *M.C.*, M.D., Ch.B. ... ... ... | "Wargrave," Clevedon. |
| *MacWatters, J. C., *M.B.E.* ... ... ... ... | Almondsbury, near Bristol. |
| *McKenzie, W. G., *M.C.* ... ... ... ... ... | 101 Coronation Road, Bristol. |
| *McLannahan, J. G. ... ... ... ... .. ... | The Mount, Stonehouse. |
| Marshall, Arthur L., M.B., B.A. Cantab. ... | Thornleigh, Upper Clapton, London, E.5 |
| *Mather, J. S., M.B., C.M. Aberd. ... ... ... | Lawrence Hill House, Bristol. |
| *Mathew, W. E., M.D. ... ... ... ... ... ... | 85 Coldharbour Road, Redland. |
| *Mayes, F. J. A. ... ... ... ... ... ... ... | 79 Pembroke Road, Clifton. |
| Miall, C. L'O. ... ... ... ... ... ... ... ... | Elm Hayes, Paulton, near Bristol. |
| *Milner, A. N. P. ... ... ... ... ... ... ... | 318 Stapleton Road, Bristol. |
| *Moore, C. A., M.B., M.S. Lond.... ... ... ... | 56 St. Paul's Road, Clifton. |
| *Morris, A. G. ... ... ... ... ... ... ... | 18 Westbury Park, Westbury-on-Trym. |
| Morris, L. N., M.B., Ch.B. Brist. ... ... ... | 24 All Hallows Road, Upper Easton, near Bristol. |
| *Morris, Mary E. H., M.D. Lond. ... ... ... | 19 Gay Street, Bath. |
| *Morton, C. A. ... ... .. ... ... ... ... ... | 14 Vyvyan Terrace, Clifton. |
| *Mumford, W. G., M.B. Lond. ... ... ... ... | 18 The Circus, Bath. |
| *Myles, G T. ... ... ... ... ... ... ... ... | 7 Apsley Road, Clifton. |

| | |
|---|---|
| *Neild, Newman, M.B., B.Ch. Vict. ... ... ... | 9 Richmond Hill, Clifton. |
| *Newnham, W. H. C., M.B., M.A. Cantab. ... | 3 Lansdown Place, Victoria Square, Clifton. |
| *Nichols, F. C., *M.C.*, M.B., Ch.B. Brist. ... | 38 Whiteladies Road, Clifton. |
| *Nixon, H. C., M.D. Edinb. ... ... ... ... | 5 Queen's Parade, Bath. |
| *Nixon, J. A., *C.M.G.*, M.D. Cantab. ... ... | 7 Lansdown Place, Clifton. |
| *Norgate, R. H. .. ... ... .. ... ... ... | Southmead Infirmary, Bristol. |

| | |
|---|---|
| Oppenheimer, Son & Co. Ltd. ... ... ... | 179 Queen Victoria Street, London, E.C.4. |
| *Ormerod, H. Lawrence, M.D., B.Ch. R.U.I. | 2 Henleaze Road, Westbury-on-Trym Bristol. |
| Orr-Ewing, H. J. *M.C.*, M.D. Lond. ... ... | English Mission Hospital, Jerusalem. |

/

### LIST OF SUBSCRIBERS.

| | |
|---|---|
| *PARKER, GEORGE, M.D., M.A. Cantab. ... ... | 14 Pembroke Road, Clifton. |
| *PARKES, J. A., M.B., Ch.B. Liverpool. ... .... | 2 Effingham Road, Bristol. |
| PARKINSON, Major G. S., *D.S.O.*, R.A.M.C.... | R.A.M.C. Mess, Grosvenor Place, London, S.W. 1. |
| *PARSONS, H. F. ... ... ... ... ... ... ... ... | The Heath, Hazelwood Road, Sneyd Park, |
| PARSONS, Sir J. H., M.B., B.S., D.Sc. Lond... | 54 Queen Anne Street, London, W. |
| PATERSON, DONALD ROSE, M.D., C.M. Ed. ... | 15 St. Andrew's Crescent, Cardiff. |
| *PHILLIPS, J. R. P., *C.B.E.* ... ... ... ... ... | Northwoods, Winterbourne. |
| *POLLARD, J., M.B., B.Ch., B.A.O. ... ... ... | 52 Cumberland Road, Bristol. |
| POWELL, J. J., M.D. Lond. ... ... ... ... ... | 2 Gloucester Road, Bishopston, Bristol |
| PRICE, D. T. M.B. Lond. ... ... ... ... ... | Castle Cary, Somerset. |
| *PRICHARD, ARTHUR W. ... ... ... ... ... ... | 6 Rodney Place, Clifton. |
| *PRIDIE, H. H., M.B., C.M. Ed. ... ... ... | 17 Oakfield Road, Clifton. |
| *PROWSE, ARTHUR B., M.D. Lond. ... ... ... | 5 Lansdown Place, Clifton. |

| | |
|---|---|
| *RAYNER, D. C. ... ... ... ... ... ... ... | 9 Lansdown Place, Clifton, Bristol. |
| *RENTON, R. A. S., *M.C.*, M.D. Durh.... ... ... | Tortworth House, Clevedon. |
| RITCHIE, THOMAS P. ... ... ... ... ... ... ... | 27 Oakfield Road, Clifton. |
| *ROBERTSON, D., M.B. Ch.B. Edin. ... ... ... | 205 Wells Road, Knowle. |
| *ROGERS, BEATRICE, ... ... ... ... .. ... | Richmond Hill, Clifton. |
| *ROGERS, B. M. H., M.D., B.Ch., B.A. Oxon. ... | 14 Mortimer Road, Clifton. |
| *ROLFE, R., B.A., M.B., B.C. Cantab. ... ... | Park House, St. Andrews Road, Avonmouth. |
| *ROSE, F. H. ... ... ... ... ... ... ... ... ... | 8 Chantry Road, Clifton. |
| *RUTHERFORD, J. M., M.B., C.M. Edin. ... ... | Brislington House, near Bristol. |

| | |
|---|---|
| *SALISBURY, WALTER, M.D., M.S. Lond. ... | Glanford, Oswold Road, Scunthorpe, Lincs. |
| *SHAW J. E., M.B., C.M., Edinb. ... ... .... | 23 Caledonia Place Clifton. |
| *SHORT, A. R., M.D., B.S., B.Sc. Lond. ... .. | 69 Pembroke Road, Clifton. |
| *SHORT, L. J., M.D., B.S. Lond., M.D. Brist. ... | Grove Park House, Weston-super-Mare. |
| *SMITH, D. MUNRO ... ... ... ... ... ... ... ... | 479 Fishponds Road, Bristol. |
| *SMITH, G. COCKBURN, M.D. Brux. ... ... ... | 12 South Road, Newton Abbot. |
| SMITH, REV. REGINALD, M.A. Cantab. ... ... | The Vicarage, Walpole St. Andrew, Norfolk. |
| *SMITH, W. ALEXANDER M.B., M.A. Oxon. ... | 70 Pembroke Road, Clifton. |
| *SMYTH, RICHARD, M.A., M.D. Dub. ... ... | Burnley, Westbury-on-Trym. |
| *SMYTHE, H. J. D., *M.C.*, M.B., M.S. ... ... | 72 St. Paul's, Road, Clifton. |
| SOCIETY, CARDIFF MEDICAL (*Hon. Lib.*, Queen Street, Cardiff). | |
| *STOCK, W. S. V., M.B., B.S. Lond. ... ... ... | 1 Mortimer Road, Clifton. |
| *STATHAM, R. S. S., *O.B.E.*, M.D., M.Ch. Brist. | 24 College Road, Clifton. |
| *STEWART, A. L., D.M., D.Ch. Brux. ... ... | 31 Howard Road, Eastville. |
| STUART, AGNES, M.B., Ch.B. ... ... ... ... | Raleigh Road, Bedminster, Bristol. |
| *SWAIN, JAMES, *C.B.*, *C.B.E.*, M.D., M.S. Lond. | 4 Victoria Square, Clifton. |
| *SWAYNE, W. CARLESS, M.D. Lond. ... ... ... | 2 Clifton Park, Clifton. |
| *SYMES, J. O., M.D. Lond. ... ... ... ... ... | 71 Pembroke Road, Clifton. |

### LIST OF SUBSCRIBERS.

| | |
|---|---|
| *Tasker, D. G. C., M.B., M.S. Lond. | 16 All Saints Road, Clifton. |
| *Tate, Colonel G. W. *C.M.G.*, *D.S.O.*, M.B., B.Ch. Dub. | 8 Park Mansions, Park Street, Bath. |
| Taylor, A. L. | The Royal Infirmary, Bristol. |
| *Taylor, H. N., M.A. Cantab., M.D. Dub. | Hillside, Ebbw Vale. |
| *Taylor, James | 36 Alma Road, Clifton. |
| *Temple, G. H., M.B., C.M. Ed. | Ailanthus, Weston-super-Mare. |
| Templeton, W. L., M.B., Ch.B. Glasgow | 143 Cheltenham Road, Bristol. |
| *Terry, C. H., M.A., M.B., B.Ch. Oxon. | 2 Gay Street, Bath. |
| Thin, James | 54 & 55 South Bridge, Edinburgh. |
| *Thomas, J. D., M.B. Cantab. | Northwoods, Winterbourne. |
| *Todd, A. T., *O.B.E.*, M.B., Ch.B. Edin. | Clayton, Clifton Park, Clifton. |
| *Trist, J. R. R., *M.C.* | 32 Pembroke Road, Clifton. |
| *Tryon, V., M.B., Ch.B. Brist. | 3 Harley Place, Clifton. |

| | |
|---|---|
| *Vickery, W. H. | 1 Trewartha Park, Weston-super-Mare. |

| | |
|---|---|
| *Walker, Cyril H., M.B., B.A. Cantab. | 8 Oakfield Road, Clifton. |
| *Wallace, John, *O.B.E.*, M.B., C.M. Edin. | Lauriston, Weston-super-Mare. |
| *Wallace, J. T., M.B., B.Ch., B.A. R.U.I. | 48 Coronation Road, Bristol. |
| *Walters, C. F. | 5 Mortimer Road, Clifton. |
| *Wanklyn-James, C. W., *O.B.E.* | 3 Mortimer Road, Clifton. |
| *Warren, R., M.A., M.D. Oxon. | 1 Royal Terrace, Weston-super-Mare. |
| *Waterhouse, R., M.D. Lond. | 25 Circus, Bath. |
| *Watson-Williams, P., M.D. Lond. | 2 Rodney Place, Clifton. |
| *Watson-Williams, E., *M.C.*, M.B., B.Ch., B.A. Cantab. | 12 Victoria Square, Clifton. |
| *Whicher, A. H. | 115 Queen's Road, Clifton. |
| Whitehouse, H. Beckwith, M.S. | 52 Newhall Street, Birmingham. |
| Wigan, C. A., M.D. Durh., J.P. | Deepdene, Portishead. |
| Wigmore, J., M.D. R. U. I. | 16 Queen Square, Bath. |
| *Wilkinson, S. H., M.D. Edin. | Pensions Hospital, Ashton Court. |
| *Willcox, J. W. J., M.B., B.S. Lond. | Glastonbury. |
| Willcox, R. Lewis | 97 London Road, Salisbury. |
| *Williams, E. C., M.B., B.C., B.A. Cantab. | 159 Whiteladies Road, Clifton. |
| *Williams, G. C., B.A. Cantab. | Ham Green, Pill. |
| Williams, H. Egerton | The Mount, Newport, Mon. |
| *Williams, L. H., M.D. Durh. | Thornbury, Glos. |
| Williams, S. R., M.B. Lond. | Buckfastleigh, Devon. |
| *Wills, W. K., M.B., B.C. Cantab. | 19 Whiteladies Road, Clifton. |
| *Windsor-Aubrey, H. W. | 79 Coronation Road, Bristol. |
| *Wood, Duncan | 5 Eaton Crescent, Clifton. |
| Wood, W. V., *M.C.* | Henley Lodge, Yatton, Som. |
| Woodcock, O. H., M.D., Ch.B. Man. | Dunmurray, Sneyd Park, Clifton. |
| Wright, A. J., M.B., B.S. Lond. | 14 Victoria Square, Clifton. |

### The Library of the Bristol Medico-Chirurgical Society.

*The following donations have been received since the publication of the List in March,* 1923.

*January,* 1924.

| | | |
|---|---|---|
| Mr. Hey Groves (1) | .. .. .. .. | 4 volumes. |
| Medical Research Council (2) | .. .. .. | 3 ,, |
| Mr. Rendle Short (3) | .. .. .. .. | 1 volume. |
| The Surgeon-General, United States Army (4) | .. | 1 ,, |
| J. Wright & Sons (5) | .. .. .. .. | 1 ,, |

Unbound periodicals have been received from Dr. Carey Coombs and Mr. Hey Groves.

### THE ONE HUNDRED AND EIGHTH LIST OF BOOKS.

The figures in round brackets refer to the figures after the names of the donors. The books to which no such figures are attached have been received through the *Journal.*

| | | |
|---|---|---|
| **Borradaile, L. A.** .. | *Elementary Zoology* .. .. .. .. | [1923] |
| **do.** .. | *Manual of Zoology* .. .. .. 4th Ed. | [1923] |
| **Bidou, G.** .. .. | *Nouvelle méthode d'Appareillage des Impotents* | 1923 |
| **Cammidge and H. A. Howard, P. J.** | *New Views on Diabetes Mellitus* .. .. .. .. .. .. .. .. .. | [1923] |
| **Campbell, H.**.. .. | *Man's Mental Evolution* .. .. .. .. .. | 1923 |
| **Capparoni, P.** .. | *Magistri Salernitani Nondum Cogniti* .. .. | 1923 |
| *Catalogue (Index-) of the Library of the Surgeon-General's Office,* | *United States Army* (4) 3rd Ser., Vol. III. | 1922 |
| **Choyce, C. C.** [Ed.] | *System of Surgery.* 3 vols. .. [2nd Ed.] | 1923 |
| **Christiansen, V.** .. | *Les Tumeurs du Cerveau* .. .. .. (1) | 1921 |
| **Coke, F.**.. .. .. | *Asthma* .. .. .. .. .. .. .. .. .. | 1923 |
| **Cope, Z.** .. .. | *Early Diagnosis of the Acute Abdomen.* 2nd Ed. .. .. .. .. .. .. .. .. .. .. | [1923] |
| **Dally, J. F. H.** .. | *High Blood Pressure* .. .. .. .. .. .. | 1923 |
| **Donaldson, R.** .. | *Practical Morbid Histology* .. .. .. .. | 1923 |
| **Fowler, Sir J. K.** .. | *Problems in Tuberculosis* .. .. .. .. | [1923] |
| **Glynn, E. E.**.. .. | *Observations on Pneumonia and Empyemata* (2) | 1923 |
| **Gordon, A. K.** .. | *Hæmatology* .. .. .. .. .. .. .. .. | 1923 |
| **Gould, Sir A. P.** .. | *Surgical Diagnosis* (Ed. by E. P. Gould) 6th Ed. .. .. .. .. .. .. .. .. .. | 1923 |
| **Graham, G.** .. .. | *Pathology and Treatment of Diabetes Mellitus* | [1923] |

**Hawkins, E.** .. .. *Medical Climatology* .. .. .. .. .. .. 1923
**Hayem, G.** .. .. *L'Hématoblaste* .. .. .. .. .. .. .. .. .. 1923
**Hehir, Sir P.** .. *The Medical Profession in India* .. .. 1923
**Hoefftcke, C. A.** .. *Ambulatory Treatment of Fractures* .. .. 1923
**Howard, P. J. Cammidge and H. A.** *New Views on Diabetes Mellitus* [1923]
**Hutchinson, J.** .. *Hernia* .. .. .. .. .. .. .. .. .. .. .. [1923]
**Hutton, I. E.** .. *Hygiene of Marriage* .. .. .. .. .. .. 1923
**Jones and R. W. Lovett, Sir R.** *Orthopedic Surgery* .. .. .. 1923
**Jordan, A. C.** .. *Chronic Intestinal Stasis* .. .. .. .. [1923]
*Kata-thermometer* (*The*) *in Studies of Body Heat* (2) .. .. .. 1923
**Kenwood, L. C. Parkes and H. R.** *Hygiene and Public Health.* 7th Ed. .. .. .. .. .. .. .. .. .. .. 1923
**Kerr, J. M. M.** [*et al.*]. *A Combined Text-Book of Obstetrics and Gynæcology* .. .. .. .. .. .. .. .. 1923
**Kingscote, E.** .. *Movement in Organic Disease* .. .. .. 1923
**Lacassagne, A.** .. *A Green Old Age* (Trans. by H. Wilson) .. 1923
*Laennec's De l'Auscultation médiate.* Selected passages (Ed. by Sir W. H. White) .. .. .. .. .. .. .. .. 1923
**Lejars, F.** .. .. *Urgent Surgery* (Trans. by W. S. Dickie and E. Ward) .. .. .. .. .. .. .. .. 3rd Ed. 1923
**Lovett, Sir R. Jones and R. W.** *Orthopedic Surgery* .. .. .. 1923
**McKenzie, D.** .. *Aromatics* .. .. .. .. .. .. .. .. .. .. .. 1923
**Mackenzie, Sir J.** .. *Angina Pectoris* .. .. .. .. .. .. .. .. [1923]
**Mackenzie, Sir J. and J. Orr.** *Diagnosis and Treatment in Heart Affections* .. .. .. .. .. .. .. 2nd Ed. [1923]
**Martin and H. H. Weir, H.** *Medical Practice in Africa and the East* 1923
**Milligan and W. Wingrave, W.** *Diseases of the Ear* .. .. .. 1923
**Monrad-Krohn, G. H.** *Clinical Examination of the Nervous System,* 2nd Ed. .. .. .. .. .. .. .. .. .. .. 1923
**Montet, C. de** .. *Primary Problems of Medical Psychology* .. [1923]
**Morley, A. S.** .. *Hæmorrhoids* .. .. .. .. .. .. .. .. .. [1923]
**Mottram, V. H.** .. *Manual of Histology* .. .. .. .. (3) [1923]
**Mummery, P. L.** .. *Diseases of the Rectum and Colon* .. .. 1923
**Orr, Sir J. Mackenzie and J.** *Diagnosis and Treatment in Heart Affections* .. .. .. .. .. .. .. 2nd Ed. [1923]
**Osborn, H.** .. .. *Golden Rules of Dental Mechanics* [2nd Ed.] 1923
**Parkes and H. R. Kenwood, L. C.** *Hygiene and Public Health* 7th Ed. 1923
**Poulsson, E.** .. .. *Pharmacology and Therapeutics* (Ed. by W. E. Dixon) .. .. .. .. .. .. .. .. .. .. .. 1923
**Power and C. J. A. Thompson, Sir D'A.** *Chronologia Medica* .. 1923
*Reprints from the Department of Experimental Surgery, New York University,* Vol. III. .. .. .. .. .. 1920–22
*Rickets in Vienna, Studies of,* 1919–22 .. .. .. .. .. .. (2) 1923
**Schall, W. E.** .. *X-rays : Origin, Dosage and Application* .. 1923
**Schröder, R.** .. *Lehrbuch der Gynäkologie* (5) .. .. .. 1922
**Stark, A. C.** .. .. *An Index to General Practice* .. .. .. 1923
**Stopes, M. C.** .. *Contraception* .. .. .. .. .. .. .. .. .. 1923

**Thompson, Sir D'A. Power and C. J. A.** *Chronologia Medica* .. 1923
**Thomson, D.** .. *Gonorrhœa* .. .. .. .. .. .. .. .. .. .. [1923]
**Thorne, L. T.** .. *"Nauheim" Treatment* .. .. .. 6th Ed. 1923
**Walker, C. E.** .. *Theories and Problems of Cancer* .. .. .. 1923
**Walker, G.** .. .. *Venereal Disease in the American Expeditionary Forces* .. .. .. .. .. .. .. .. .. .. [1922]
**Walker, K. M.** .. *Diseases of the Male Organs of Generation* .. [1923]
**Wallace, J. S.** .. *Oral Hygiene* .. .. .. .. .. .. .. .. .. 1923
*War, Surgery of the* 2 vols. .. .. .. .. .. .. .. .. .. (1) 1922
**Whiteford, C. H.** *Surgical "Don'ts" (and "Do's")* .. .. [1923]
**Wingrave, W. Milligan and W.** *Diseases of the Ear* .. .. .. 1923

TRANSACTIONS, REPORTS, JOURNALS, etc.

American Association of Genito-Urinary Surgeons, Transactions of the .. .. .. .. .. .. .. .. .. .. .. Vol. XV. 1922
American Laryngological Association, Transactions of the .. .. 1919
Archiv für klinische Chirurgie (1) .. .. .. .. .. Bd. CXXIII. 1923
Association of American Physicians, Transactions of the Vol. XXXVII. 1922
College of Physicians of Philadelphia, Transactions of the Vol. XLV. 1922
London Dermatological Society, Transactions of the .. .. .. 1923
Medical Annual, The .. .. .. .. .. .. .. .. .. .. .. .. 1923
Ophthalmological Transactions .. .. .. .. .. .. Vol. XLIII. 1923
Smithsonian Report for 1921 .. .. .. .. .. .. .. .. .. .. 1922
Studies from the Department of Pathology, College of Physicians and Surgeons .. .. .. .. .. .. .. .. .. .. Vol. XVIII. 1921–22

### Obituary.

### R. G. Poole Lansdown, M.D., B.S.,

### Local Medical Notes.

Examination Results.—Students of the University have recently passed the following examinations:—

University of London.—M.D. (*Midwifery and Diseases of Women*): P. J. Veale (University Medal).

M.B., B.S.—*Second Examination:* A. S. Cox, Mildred B. Harvey. *Final Examination:* Lydia M. Houghton. (Group II.): W. H. Royal.

M.R.C.S., L.R.C.P. Lond.—J. R. Nicholson-Lailey, Hilda R. Dutton, E. G. Bradbeer, F. Langford, H. M. Golding, C. F. Wilson, A. E. Sherwell, H. Taylor, R. C. Hatcher, E. F. King, P. Joscelyne.

Conjoint Examining Board.—*Medicine:* D. E. Joscelyne, J. E. Hamilton, E. V. Butler, J. L. Griffin. *Surgery:* N. J. England. *Midwifery:* N. J. England, R. N. Ealand, G. Dunn, L. Dunn, W. O. Evans.

L.D.S., R.C.S.—C. Morgan.

L.M.S.S.A.—Mary E. Somers.

### *PUBLICATIONS RECEIVED.*

---

From Baillière, Tindall and Cox, London:

*An Introduction to Surgical Urology.* By William Knox Irwin, M.D., F.R.C.S. Edin. 1923. Price 7s. 6d. net.

*Man's Mental Evolution, Past and Future.* By Harry Campbell, M.D. 1923. Price 3s. 6d. net.

From J. and A. Churchill, London:

*Transactions of the Ophthalmological Society of the United Kingdom.* Vol. XLIII. 1923.

From William Heinemann Ltd., London:

*Psycho-analysts analysed.* By P. McBride, M.D., with introduction by Sir H. Bryan Donkin, M.D. 1924. Price 3s. 6d. net.

From E. and S. Livingstone, Edinburgh:

*The Insulin Treatment of Diabetes Mellitus.* By P. J. Cammidge, M.D. 1924. Price 6s. net.

From Oxford Medical Publications, London:—

*Pathology and Treatment of Diabetes Mellitus.* By George Graham, M.A., M.D. [1923.] Price 6s. net.

*New Views on Diabetes Mellitus.* By P. J. Cammidge, M.D., and H. A. H. Howard, B.Sc. [1923.] Price 21s. net.

From "The Prescriber" Offices, Edinburgh:

*Incompatibility in Prescriptions.* By Thos. Stephenson, D.Sc. 1924. Price 1s. 6d. net.

From John Wright and Sons Ltd., Bristol:

*Two Lectures on Gastric and Duodenal Ulcers.* By Sir Berkeley Moynihan. 1923. Price 2s. 6d. net.

---

*For the health worried.*

BENGER'S Food for INFANTS, INVALIDS & the AGED.

Benger's Food gives digestive rest with full nourishment, and doctors agree that this in itself is one of the finest nerve restoratives.

Sold in tins by all Chemists. Prices: 1/4; 2/3; 4/-; 8/6

### THE Hospital and Health REVIEW

A JOURNAL FOR EVERYONE INTERESTED IN PUBLIC HEALTH, PREVENTIVE AND CURATIVE MEDICINE AND HOSPITAL AND INSTITUTIONAL WORK.

*Established 1886.*

Published MONTHLY, Price 6d.

THE HOSPITAL AND HEALTH REVIEW includes within its sphere the various aspects of PUBLIC AND PERSONAL HEALTH, PREVENTIVE AND CURATIVE MEDICINE, HOSPITAL AND INSTITUTION WORK, AND THE GENERAL WELL-BEING OF THE PEOPLE. These subjects of truly vital concern are dealt with in plain, and as far as possible non-technical, language, so that they may be understood, and consequently of *service to everyone* interested in these matters.

Price 6d. of all Booksellers and Newsagents, or 7½d. post free of the Manager, "The Hospital and Health Review," 28/29 Southampton Street, Strand, London, W.C. 2.

